# Peer-review Blinded Assay Test (P-BAT): a framework for trustless laboratory quality assurance for state-regulated cannabis markets

**DOI:** 10.1186/s42238-025-00261-3

**Published:** 2025-01-21

**Authors:** Stuart Procter, Grayson L. Baird, Jason Iannuccilli

**Affiliations:** PureVita Labs LLC, 153 James P Murphy Ind Hwy , West Warwick, 02893-2382 United States

**Keywords:** Quality assurance, Transparent, Trustless, Cannabis, Laboratory fraud

## Abstract

The purpose of laboratory testing in the cannabis industry is to ensure public safety by preventing products that exceed hazardous limits of contaminants from reaching consumers, and to provide consumers with transparent and accurate label information so that they can make informed decisions when purchasing and using products. However, cannabis testing does not exist in a vacuum of incentives—some incentives exist that are in direct conflict with what is best for consumers. For example, cultivators and distributors will prefer to use the services of laboratories that find the highest THC concentrations or lowest contaminant concentrations, regardless of the accuracy of their testing results. Laboratories will, therefore, be incentivized to serve the cultivators and distributors over the end consumer. The present essay proposes a framework for quality assurance that combats these perverse incentives. The following proposed framework called the Peer-review Blinded Assay Test (P-BAT), is a validation process where each laboratory tests products from competing labs and their own lab, but in a blinded fashion to ensure that the label values of said products and the labs that produced said labels, are unknown. This system of blinded self-review and peer-review is designed to be cost-efficient, transparent, nearly self-funded, can be implemented in any state with two or more laboratories, and most importantly, it is trustless—there is no need to trust the behavior of any one actor or laboratory to serve as a “gold standard”. While the primary objective of this process is to focus on laboratory performance, it will also highlight other common problems in the industry such as product adulteration by distributors and poor storge practices. Data from P-BAT should be publicly available so consumers can make informed decisions about their purchases based on the quality data derived from P-BAT. Doing so would further incentivize laboratories to serve and be accountable to the end consumer instead of cultivators and distributors.

## The Problem

Each state that regulates a cannabis industry is responsible for implementing a set of analytical testing regulations with which laboratories need to comply. The purpose of laboratory testing in the cannabis industry is twofold: 1.) To ensure consumer safety by preventing products that exceed hazardous limits of contaminants (established by policy) from reaching consumers; 2.) To provide consumers with transparent and accurate label information so they can make informed decisions about product purchases and use. If laboratory testing fails to operate as expected, it becomes difficult to differentiate between the legal market and the illicit market in terms of product safety. This situation can also mislead consumers with inaccurate information and expectations. Therefore, the cornerstone of a functioning, reputable cannabis industry lies in high-quality, correctly labeled, safe products reaching the public. This requires a laboratory network that has the capacity to meet the industry’s needs while maintaining a high degree of integrity, analytical accuracy, and precision. Consequently, properly functioning laboratories represent the gateway to a prosperous, legitimate market as they are the only filter that defines what products and information reach the general public via thelegal market.

Although laboratory testing to ascertain the safety and quality of a product should be straightforward and objective in theory, this is not necessarily the case in practice, as recently reported on February 20, 2024, in *The Wall Street Journal* (Armour 2024). Laboratory testing does not exist in a vacuum of incentives—some incentives exist that directly conflict with what is best for consumers, an unintended consequence of testing in accordance with established thresholds that is well documented in economics and known as Goodhart’s Law (Muller 2018). Goodhart’s Law is the notion that when a measured threshold becomes a target, it ceases to be a good measure. In other words, once a metric or indicator is used as a target for decision-making or evaluation, it can lose its effectiveness as a reliable measure because people and systems may manipulate their behavior to meet the target rather than focusing on the underlying goal of the target.

For example, suppose certain labs tend to find higher Tetrahydrocannabinol (THC) concentrations than other labs. In that case, cultivators will be incentivized to use those labs over the others since retail establishments often use minimum thresholds for THCconcentration when determining the wholesale value of cannabis flower purchased directly from cultivators. Similarly, if certain labs are less likely to detect contaminants than others, cultivators are incentivized to send their samples to these labs. This choice is driven by the desire to bypass any potential barriers to market entry posed by the detection of contaminants in their products. Although some forms of product contamination can be remediated, these processes usually add additional overhead costs to production and risk creating the illusion of an inferior product in the eyes of the consumer. These perverse incentives undermine the purpose of independent lab testing, which aims to establish an objective and accurate analytical process that ensures all products undergo the same level of regulatory quality control.

At present, the greater public opinion is that high THC concentration and lack of product remediation are the most important attributes that determine cannabis product quality. Retail dispensaries currently control procurement and market availability of products to consumers and price products based on THC potency thresholds and whether a product has undergone remediation practices. Therefore, cultivators will be incentivized to use some labs over others based on the results they achieve instead of the accuracy of those results, leading to a phenomenon known as “Lab shopping.”(Paulson et al. 2022; Schwabe et al. 2023; Smith 2018).

“Hero sampling” is another negligent or fraudulent practice that has disrupted the integrity of the cannabis market and led to the widespread inaccuracy of product labels. Hero sampling refers to practices where a premium sample is selected by either a lab or a cultivator using non-randomized methods that do not adhere to statistical sampling, resulting in the test sample not being adequately representative of the product that will ultimately be sold to the public bearing the inaccurate (and usually inflated) potency levels on its label. (Paulson et al. 2022; Schwabe et al. 2023; Smith 2018; Toth 2022) Labs are therefore incentivized to compete to achieve "better" results for their industry customers (cultivators), not necessarily more accurate results for consumer safety and product labeling. This leads to general inflation of THC and other cannabinoid potency numbers and an underestimation of hazardous contaminants. Consequently, these practices undermine the integrity and legitimacy of the legal cannabis industry as a whole (Black 2021; Cappetta 2022). Therefore, the policing of laboratory practices should remain the highest priority of regulatory agencies to promote a legitimate legal market where labs prioritize consumer safety, not necessarily the needs of their industry customers.

Overall, most states have fairly rigorous regulations to govern the industry, but they lack the required resources for oversight and enforcement of those regulations. Without effective policing measures and the required resources to oversee such quality assurance programs, testing laboratories find themselves under constant pressure from their customers (cultivators and product manufacturers) to pass products that do not meet regulatory safety thresholds or generate inflated potency numbers to compete on the open market. A lab not cooperating with industry customers in this way will incentivize them to take their business elsewhere. There are also many ways in which a laboratory could intentionally manipulate testing results, by using poor analytical practices and suboptimal equipment, by altering data after it is collected, or through a combination of these fraudulent practices, which can be accomplished in clandestine ways that are virtually impossible for an external regulating agency to detect.

Due to these perverse incentives, a problematic situation arises: in the absence of an external quality assurance system, a laboratory could face adverse consequences for adhering to good practices, utilizing quality equipment, and providing accurate results. Conversely, another laboratory might be rewarded for employing poor practices, using substandard equipment, and producing inaccurate results. As Donald Land, a chemistry professor at the University of California Davis, highlighted in The Wall Street Journal, " Honest labs in many markets could not get market share because they would not cheat … they are paid by the growers, and the growers can decide which labs to go to. It’s so easy to cheat and get away with it if regulators aren’t on top of it.” (Armour 2024). Clearly, state-imposed taxation and fines cannot prevent hero sampling and lab shopping as such disincentives only work when a monitoring framework exists to detect such behaviors. But what are some models that are used to evaluate labs?

- Spiked Sample Model. One method of evaluating a single lab’s accuracy is to use “Spiked Samples,” which is to provide a lab a purposefully adulterated sample. Here, the objective is to compare the lab’s result with the level of adulteration for said sample.
- Vanilla Sample Model. A second method of evaluating a single lab’s accuracy is to use a pre-characterized sample where the properties of the sample are known. This uses the same methods as spiked samples, but instead with a non-adulterated sample.
- Proficiency Testing Programs. A proficiency testing (PT) program is a process that evaluates a laboratory's performance and accuracy by sending unknown samples to participating labs for testing. The program then grades the results using CLIA grading criteria and sends scores back to the labs to reflect how well they performed.
- Round-Robin Model. A method of evaluating multiple labs at the same time is a round-robin, in which a common sample is split up into multiple samples and distributed to different labs and results compared. Round Robins have numerous forms, they can be blinded or open, samples can either be unknown, characterized, or spiked samples.

However, all of these methods have limitations. Spiked and Vanilla Sample Models are fine for assessing an unintentional discrepancy between the state’s value and each lab’s value (e.g., due to differences of equipment and process), a question of inter-lab variability or consistency, but these could not detect intentional discrepancy (fraud) because 1.) labs would know a priori their results are being compared to a reference standard, so any deception would be easily detected, 2.) this approach does not measure actual product in the marketplace tested by labs, 3.) therefore, there is no way of comparing a lab’s result with itself and its own tested product, a question of intra-lab variability or consistency. Thus, if a discrepancy exists, its source cannot be deduced: statistically, lack of control for intra-lab variability means any discrepancy could be due to inter or intra-lab variability or a combination thereof. What is more, for methods that require a Spiked or Vanilla Sample, how “ground truth” is defined, especially when product cannot cross state lines, is difficult to establish. Regarding uncharacterized samples, most states do not have enough labs to approximate a “ground truth” via statistical analysis of a large data set.

As mentioned, for any of these options to be effective, the sample needs to be analyzed without knowledge of the testing lab. The reason is straightforward: When a lab becomes aware that they are being evaluated, that sample is likely to receive different treatment compared to others, whether consciously or unconsciously. This phenomenon is known as the Hawthorne effect.” (Mayo 1949) Testing a lab without the lab being aware of the fact is particularly difficult to achieve, especially in states where samples are independently sampled, a model that is gaining popularity with states as they try to minimize “Hero Sampling”. Ultimately, because regulation exists in the first place, labs know that they will be evaluated. Therefore, the Hawthorne effect cannot be removed entirely, but it should be mitigated as much as possible.

A fifth option is the secret shopper model. The secret shopper model has a state agent purchase products off the shelf and then analyze them, looking for instances of discrepancies between the lab result and the label. Although this model prevents a Hawthorne effect, any discrepancy observed between a product’s label and the lab results used by a secret shopper may or may not indicate a true discrepancy because one or both labs may be in error. And even if the lab used by the secret shopper is a state-funded lab, a secret shopper would need to accumulate large amounts of data from the same distributor for the same product using the same lab in order to show evidence of systematic differences from a given lab, statistically. But this would require a state to run its own testing lab.

## A Solution

A challenge for state regulators in overseeing the cannabis industry is the need for effective solutions and resources to prevent "Lab Shopping", "Hero sampling” and other forms of product adulteration. It is cost-prohibitive and inefficient for a state to have its own independent lab to test products off the shelf. What is more, because of a Hawthorne effect, labs can only be evaluated when they are unaware that they are being evaluated, which can be very difficult to implement, relies on trust of all parties to execute, and can be difficult to know when protections to prevent unblinding have failed. Therefore, the solution to these problems needs to be a quality assurance framework that disincentivizes “Lab shopping” and “Hero sampling,” reduces the Hawthorne effect, and is decentralized but also scalable in any applicable state. The following proposed framework, what we call hereon the Peer-review Blinded Assay Test (P-BAT), is a validation process where each laboratory tests products from competing labs and their own lab, but in a blinded fashion as to ensure that the label values of said products, and the labs that produced said labels, are unknown. This system of blinded self-review and peer-review is designed to be cost-efficient, transparent, nearly self-funded, can be implemented in any state with two or more laboratories, and finally, trustless—there is no need to trust the behavior of any one actor or laboratory. Furthermore, this process could implement the Hawthorne effect in a positive manner as the psychology of knowing that each COA that leaves the testing facility could at any point be tested again by peers and themselves could result in additional care and due diligence on generating accurate reproducible data. The example here is tailored for flower products, but all product types could be tested using P-BAT.

### A. The general process would be as follows assuming (for this example, 3 laboratories):

**Fig. 1:**
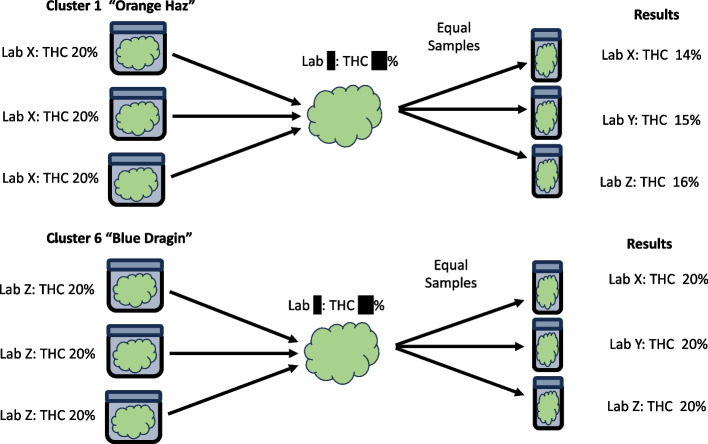
Diagram showing graphical representation of a two-stage cluster sampling design

1. Once a month, two or more state representatives (employees or contractors, defined by each state) randomly select a distributor from which to sample. These representatives randomly select a minimum of 2 product lines to test for each lab. They then randomly select 3 units of this product line to be purchased. Therefore, if 2 product lines were chosen, a total of 6 units per lab would be required. For example, a single distributor is randomly selected once a month by the state. State representatives then enter that distributor and randomly select 2 or more product lines for each lab. If there were 3 labs, then there would be 2 product lines per lab and 3 units would be randomly selected per product line for a total of 18 samples purchased (3 X 2 X 3) (see Fig. 1). Likewise, for states with 6 labs, there would be 2 product lines per lab, 6 units per product line for a total of 72 samples (6 X 2 X 6). For states with more than 6 labs, a randomized block design could be adopted where the blocks are randomized every 12 months. For example, for a state with 18 labs, 6 labs would be randomly assigned to 3 blocks, where the 6 labs in each block evaluated themselves each month. In this case, each block would have its own sampling event every month. The samples could be commandeered or purchased at a state-enforced discounted rate to cover the cost of materials. State representatives would never visit labs to collect samples.
2. The three units of the same product, from the same cultivators with the same THC content, tested from the same lab (i.e. everything held constant) obtained from the same distributor on the same day are combined into a single sample that is then equally distributed into three sterile 50 ml centrifuge tubes (see Fig. 1). Labs are therefore blinded to all product information including, name, THC content, level of contaminants, and Lab that did the testing. Each sample will receive an accession number with no other details provided. These samples could either be delivered to each lab or collected by the lab from a central location on the same day.
3. Each lab would analyze all the samples provided for the required tests, and the results reported to the state no later than 10 days after receiving the sample. In this fashion, each lab will re-analyze samples previously tested by their own facility as well as samples tested by other facilities without knowing which samples are which.
4. The data should be collated by the state, enabling the comparison of a given lab’s data to the original Certificate Of Analysis (COA) that they generated themselves, as well as the lab-to-lab results.
5. State statisticians or independent statisticians would review data to measure variability and the presence of trends over time, known as Statistical Process Control (SPC).

### B. Additional Detail of the P-BAT process (see Fig. 1)

1. For step 1, the number of samples collected would depend on the number of active labs in the state. If there were 3 active labs (as in our current example), then 6 product lines, 2 from each lab, could be examined (note, many more could be used). Sample selection could be either targeted or untargeted. For example, if discrepancies in Microbiological data were of interest, targeting COAs that have detected microbes, but still pass should be selected. It is these samples that typically exhibit the largest lab-to-lab differences, thus highlighting either fraudulent activity or bad analytical procedures. The same information could be obtained from a non-targeted sample collection, but that would require many more samples to find evidence of trends.
2. For step 2, homogenization of the clusters with appropriate equipment would be optimal; otherwise sampling error would require more samples to be collected. Simple mixing without specialized equipment will still yield the required data, but once again, more data points would be required to observe statistically meaningful trends.
3. For step 3, it may help to start with a focus on testing for THC concentration and microbiological contaminants in flower products while testing for THC concentration and pesticide residues in concentrate products. This will minimize the amount of sample material required for testing while focusing on the key areas where most discrepancies in data are currently observed between labs.
4. For step 4, the severity of a particular problem will dictate the amount of data required to detect the issue, but the benefit of this framework is that it will likely instill the same behavior exhibited in an open round-robin where a lab is aware that their data will be compared with data derived from other labs for the same sample. That is, the behavior will change based on the knowledge that every COA released out the door could undergo further scrutiny by their own hands and peer labs in the state, thus disincentivizing “Hero sampling” and “Lab shopping”. As processes improve, errors will reduce, thus requiring fewer samples.
5. For Step 5, the sampling design is a *two-stage* cluster design, as depicted in Fig. 1: The population is all products available in distributors in the state. The sampling frame is all products available in the distributor on the day the sampling is taking place. Clusters are defined as a given product line (e.g., for *Stage 1*, six different product lines are randomly selected, 2 from each lab) to be tested (e.g., “Orange Haz”, THC 20%, Tested by Lab X” would be a Cluster 1, while “Blue Dragin,” THC 20%, Tested by Lab Z would be Cluster 6). Within a cluster (product line), three individual product packages are then randomly selected (e.g., for *Stage 2*, Cluster 1, 3 packages of "Orange Haz” would be purchased; for Cluster 6, 3 packages of “Blue Dragin” would be purchased). These three packages, which are of identical product, from the same distributor, with the same label (THC content, etc.), and generated from the same lab, are then combined into one sample and equally portioned out into three separate samples to be tested by the three competing and blinded labs. The results from the blinded review are then compared with the original lab values of the product, demonstrated in Table 1. With these data, using statistical analyses with random effects, trends by lab, product, cultivator time, and distributor (to be described in the next section) can all be measured, thus informing where systematic (bias) and random (noise) variation can be reduced.

**Table 1:**
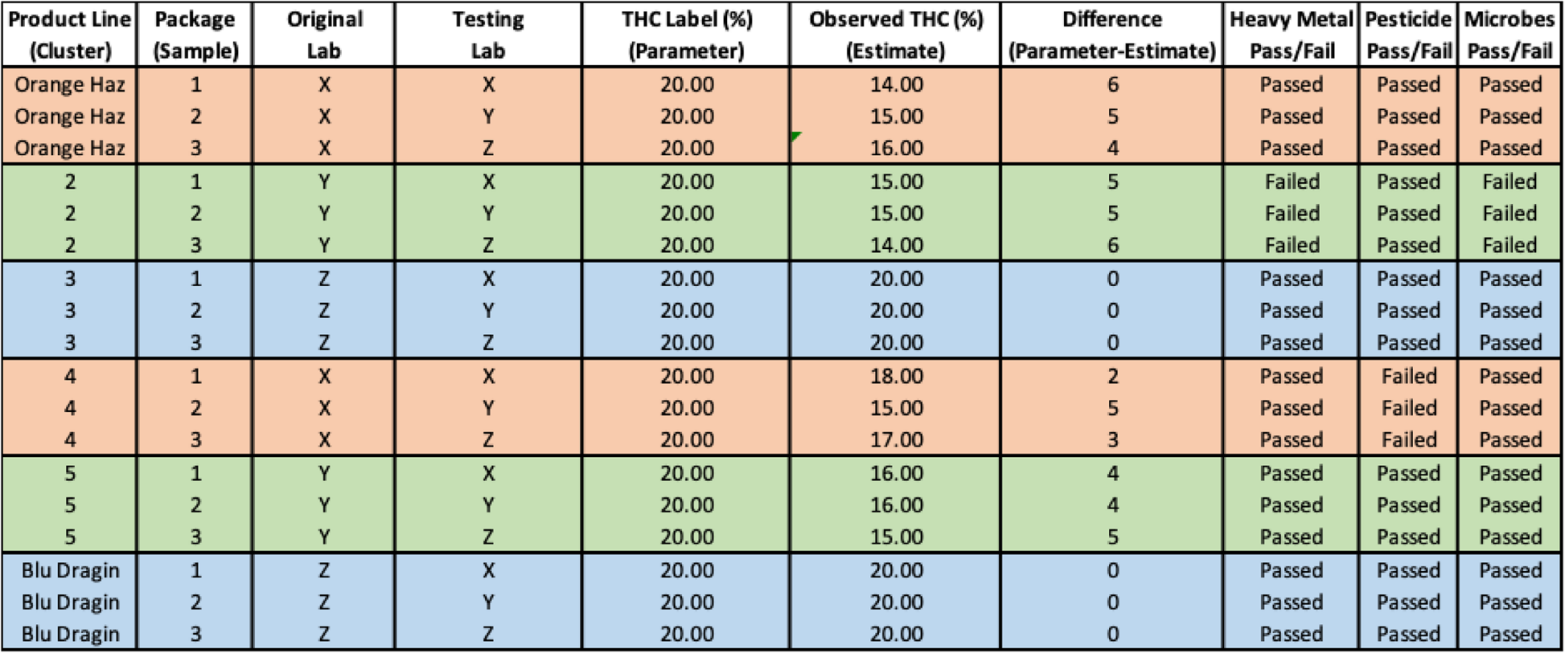
Example of 6 product lines (Clusters), 3 product packages, 3 labs concerning THC, Heavy metals, Pesticides, and Microbes

The hypothetical example in Fig. 1 and Table 1 will now be analyzed in Table 2 and interpreted between the 3 labs for 6 product lines with 3 samples for each product line. Note, this is the most basic statistical analysis possible; it is provided for illustration only and is purely hypothetical.

**Table 2:**
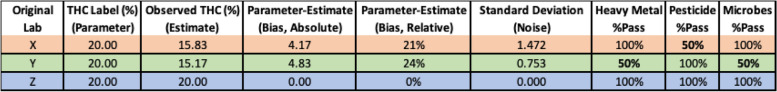
Report Card, by Lab concerning THC, Heavy metals, Pesticides, and Microbes

As can be seen in Table 2 and in detail in Table 1: A) Lab Y had the largest absolute difference 4.83) and relative difference (24% less) between the label THC and the test THC—the largest bias, 24% lower THC than advertised, B) Lab X had the second largest absolute difference (4.17) and relative difference (21%) between the label THC and test THC—21% lower THC than advertised, C) Lab Z had the lowest absolute (0%) and relative difference (0%) between the label and test THC—0%—no difference, D) Lab Z had the lowest error with a standard deviation of 0.0%—the most consistent label, E) Lab X had the highest error with a standard deviation of 1.472%—the least consistent label, F) Lab Y only passed 50% of heavy metal and 50% Microbes tests, G) Lab X only passed 50% of pesticide tests, H) Lab Z passed 100% of heavy metal, pesticide, and microbes tests.

Clearly, Lab Z has the best process, having the least bias between the label and test THC value (0% vs. 4.83% and 4.17%) and the most consistent label (0.0% vs. 1.472% and 0.753%). In addition, Lab Z passed all heavy metal, pesticide, and microbes tests. Conversely, Lab Y had the worst process, having the most bias between the label and test THC value (4.83% vs. 0% and 4.17%) and the least consistent label (1.472% vs. 0.0% and 0.753%). In addition, Lab Y passed only 50% of heavy metal and microbe tests.

**Table 3:**
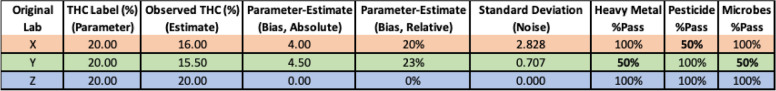
Report Card, by Lab concerning THC, Heavy metals, Pesticides, and Microbes comparing their labels and their own test results

As can be seen in Table 3 and in detail in Table 1, when comparing a Lab’s results with itself: A) Lab Y had the largest absolute difference (4.5) and relative difference (23% less) with itself for THC, B) Lab X had the second largest absolute difference (4.0) and relative difference (20%) with itself for THC, and C) Lab Z had the lowest absolute (0%) and relative difference (0%) between itself for THC. Clearly, Lab Z has the best process, having the least bias between its label and its own results.

To be clear, observed discrepancies between label values and the lab values, and values between the labs, can be due to random sampling error and measurement error—natural variations that randomly vary from sample to sample. That is, in some cases, labels will be randomly off while test results will be correct, and in other cases, labels will be correct, but test results will be incorrect; however, it is unlikely that test cases will be incorrect for all three labs (the next paragraph will explore this source of error). Over time, these results will either reveal that observed discrepancies balance out, thus being random measurement and sampling error, or they will show historical trends, thus showing systematic error (bias)—inflation or deflation of THC label values and/or underestimation of heavy metal, pesticide, or microbiological contaminants (microbes). In addition, even if bias is not observed for a particular lab, the value of the observed error can be historically compared, thus showing which labs are the most consistent (lowest error, historically) or the least consistent (largest error, historically) with their labels.

### C. Systematic error (bias) from other sources

1. *Distributor adulteration effect*. However, some sources of sampling error are not due to natural variation but instead bias, but this bias is not from the testing laboratory. The P-BAT system thus far assumes the product being tested off the shelf is the same product tested by the lab. However, it is possible that producers and retailers could cut products with cheaper, illicit, or synthetic products (Monti et al. 2022) with the aim of stretching their supply—product adulteration by distributors. Here, the adulterated results—the discrepancy between the label and the lab results—would be detected by (to continue our example) all three labs but would not be due to any fault of the testing laboratory itself but instead the distributor.
  Because distributors are chosen at random and products within distributors are chosen at random, this discrepancy would bias only the sample in question; the lab’s performance, in the long run, would be unaffected compared to the other labs (i.e., it is unlikely that one lab’s results will be consistently higher or lower in some value compared to other labs because of distributors adulteration). In this way, a “distributor effect” would increase the error of all testing labs, but the bias across the labs should be approximately equal because all labs have an equal chance of being victim to distributor adulteration, as illustrated in Tables 4, 5, and 6. What is more, because distributors are chosen at random, then the P-BAT system allows for a “distributor effect” to be detected. Specifically, the discrepancy between a label and the results from the labs should be approximately equal across all samples, as seen in Table 6 (i.e., ~ 17% vs. 20%). However, if the discrepancy from one distributor is consistently higher than the other distributors, then this is evidence of a distributor effect, as seen in Table 5 (i.e., 12.22% vs. 20.00%).

**Table 4:**
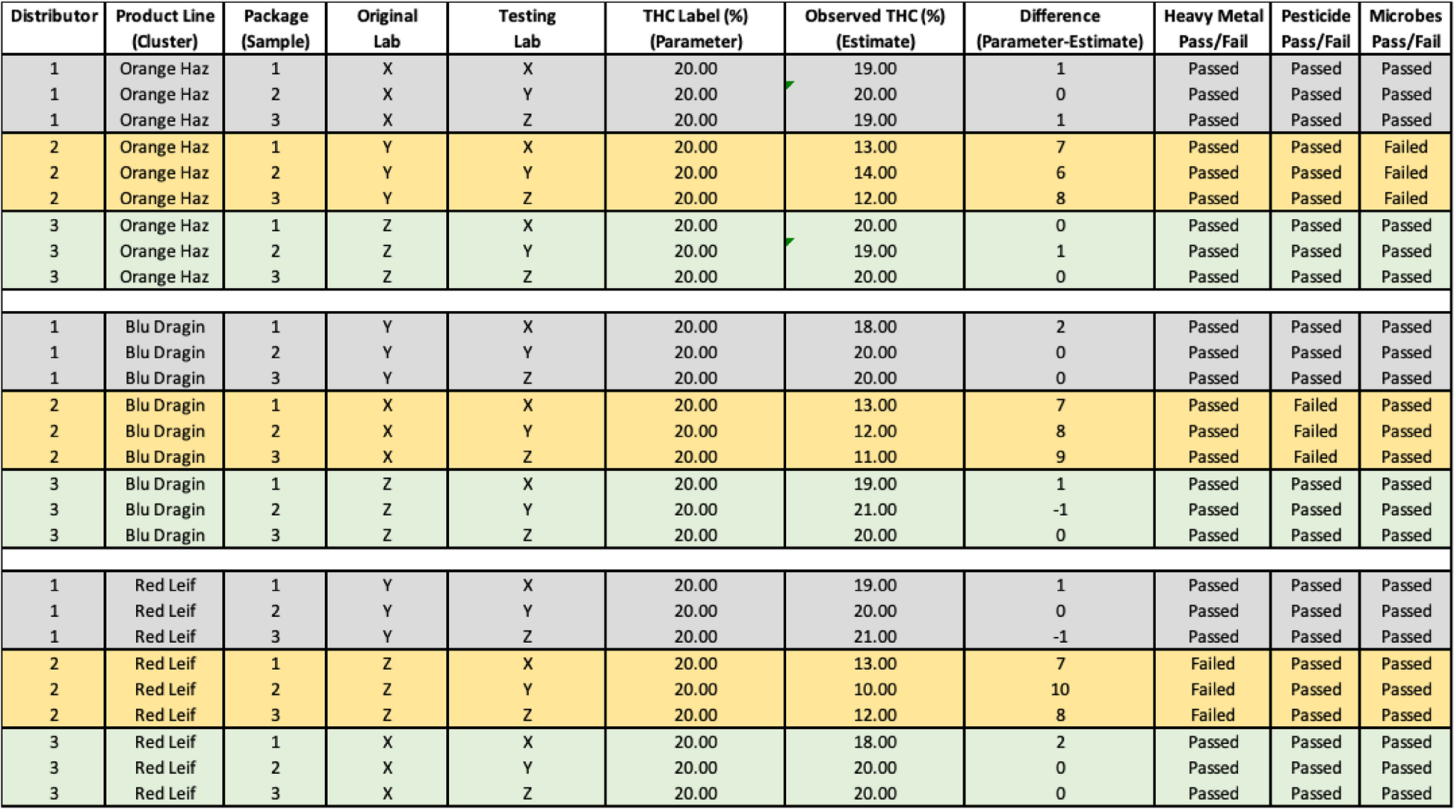
Example of 3 distributors, 3 product lines (Clusters), 9 product packages, 3 labs concerning THC, Heavy metals, Pesticides, and Microbes
  **Table 5:**
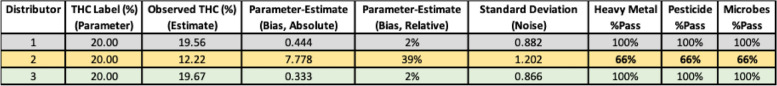
Report Card, by distributor concerning THC, Heavy metals, Pesticides, and Microbes

**Table 6:**
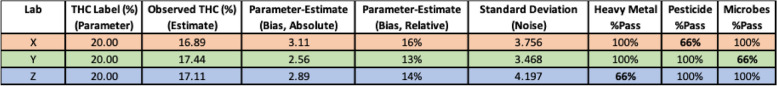
Report Card, by Lab concerning THC, Heavy metals, Pesticides, and Microbes

To prevent this source of bias, packaging could be designed to be tamperproof, not unlike regular over-the-counter medications; in this way, the producers would be incentivized to protect the quality of their product reaching the end-consumer. In addition, the threat of distributor adulteration is why products should be tested randomly from the aisles of distributors themselves and not before. That is, P-BAT provides a check on distributors, disincentivizing them to engage in adulteration of products post-testing. These preventive measures, when implemented effectively, can significantly reduce the risk of distributor adulteration, providing a sense of reassurance to the industry and consumers alike.
2. *Age effect*. Another source of sampling error is the age of the product—with age, THC content decays—an “age effect”. Because products are randomly selected when a product is tested and when it is sampled will vary from sample-to-sample, product-to-product, and thus, lab-to-lab (i.e., it is unlikely that one lab’s results will be consistently lower in THC compared to other labs because of an “age effect”). Although the discrepancy between a label and the results from the labs due to age should be approximately equal across all samples, it will nonetheless be a source of bias (and error) between the label and testing results, causing an underestimate of THC. The decay of THC due to age could be estimated and thus corrected (i.e., decay curve) to some degree if the harvest, testing, and packaging of the product were timestamped on the product packaging in a verifiable and transparent way. At the very least, now the end-consumer can anticipate age effects and make informed decisions on purchasing based on age, not unlike sell-by dates on food.
  With respect to microbiological contaminants, most, if not all, states require that products be tested for either Water Activity or Moisture content in their final form prior to packaging. Regulatory Water Activity and Moisture Content levels are set to ensure that if a product is stored correctly, microbes cannot significantly propagate after packaging. In the case of Water Activity, it is widely accepted that bacteria, yeast, and mold growth is reduced to virtually zero at water activity levels below 0.6 aw (Allen 2018). Discrepancies in microbiological contaminant levels that occur between the original regulatory testing and off-the-shelf P-BAT testing could either be due to inaccurate regulatory testing data (incompetence or fraud), faulty packaging, or poor environmental conditions under which the packaged product was stored. Either way, the P-BAT system would help identify a problem that would be worthy of additional investigation. Given the required random nature of proper sampling techniques, statistical trends would soon become obvious that may suggest whether it was a storage problem or inaccurate data generated by a lab.
3. *Unintended Technique effect.* Another source of measurement error, not due to natural variation but bias, is the use of different equipment and techniques by different labs, as mentioned earlier. Without P-BAT, labs may not even be aware that they are producing discordant results with other labs. Over time, cultivators may become aware of this bias and choose labs that are more likely to pass a product. Ironically, these labs may not realize they are the “preferred” lab without one-to-one lab comparisons as feedback. This bias poses a significant threat to the reliability and concordant validity of testing itself, as results between labs can never be comparable if they vary as a function of how they were observed (i.e. if two methods are used but can contradict each other, then how can they be valid for decision making?). For instance, using different equipment can affect the sensitivity of a test for a given method, while using different techniques can affect the selectivity of a test for a given method or any combination thereof.
  This is why it is important for states to be specific and, at the very least, consistent as to the equipment and techniques that they prescribe to ensure “apples-to-apples” for any given test (i.e., mold, pesticides, etc.). Over time, data could be collated and analyzed in a similar fashion to large scale PT programs where data is grouped by analytical technique to compare like for like methodologies between labs. Furthermore, comparing the data from different equipment and techniques over time will provide regulators with the evidence they need to identify the most reliable and reproducible technologies and techniques to achieve and codify a standardized process (Dryburgh et al. 2018; Jikomes and Zoorob 2018). Note, however, that this measurement bias should not affect the results between the label and the original lab, as in this case, the equipment and techniques are being held constant; this is the importance of blinding in the P-BAT system—certain types of discrepancies can be ruled out because labs are blinded to their own products.
  These additional sources of bias highlight that discrepancies between a label and test results can stem from various causes. Design and statistical modeling can only reveal the existence of discrepancies and may point to trends, such as “lab effects” and “distributor effects”, that are unlikely to occur by random variation alone. However, they cannot determine the reasons behind these discrepancies. In other words, discrepancies between label and test results are a necessary but not sufficient condition of evidence that fraud, manipulation, or poor practice is occurring. It is the responsibility of regulators to monitor results and, over time, when enough data has been collected, to begin determining if trends exist and if so, to investigate the cause of these trends. Over time, a lab’s results, on average, should move closer to its label value, the delta becoming smaller and smaller in value and likewise, the standard deviation for the delta should get smaller and smaller. At some point, these values will likely stabilize.

#### Limitations

There are a few limitations worth considering with the P-BAT system. First, the P-BAT framework requires more than one lab and preferably more than two labs. Another limitation is the possibility of all labs producing biased results; however, if this were to occur, since each lab is testing itself but never knows when, discrepancies between a lab and itself will likely occur. What is more, even if discrepancies do not emerge when labs are unknowingly testing themselves, discrepancies are likely to emerge between labs because they are likely to be biased in different ways for different reasons. That is, if multiple labs continuously generate the same results for every sample, it is far more likely that these labs are generating accurate rather than biased data, which is exactly what this program is meant to encourage. To be clear, most labs introduce bias in order to obtain different results from competing labs, not the same results. However, hypothetically, if all labs were to be biased and in identical ways, then only a state lab would be able to detect this.

Another limitation is each state is unique in its laws, regulatory structure, and cannabis market. Just like blind peer review is implemented differently for different journals, P-BAT will be implemented differently depending on the needs of that state, including but not limited to the size and age of the cannabis market, number of cultivators, number of distributors, and the number of testing labs in that state. Because of these factors, how a state chooses to implement and fund P-BAT will also be unique to each state. How states define areas of concern, that is, how they define statistical irregularities, investigate these irregularities, and provide disincentives for bias, will depending on lawmakers and regulators in each state, including how taxes and fines are handled. P-BAT can only provide a data collecting framework to ensure data are as valid as possible for decisions to be made; it cannot provide guidance for how these decisions should be made. In addition, as always, unintended consequences can occur, and the P-BAT system will need to be updated should these arise, and this will vary from state to state. However, unless the system is compromised through direct corruption or unidentified sources of variance in the supply-to-customer-chain, P-BAT itself should not need improvement.

Another limitation is the scope of inference. Statistics cannot be used to prove fraud. All statistics can do is provide evidence that a value is consistently higher or lower than what would be expected due to chance variation alone (honest variation)—smoke, but not fire. In an experiment, say a randomized control design, one can deduce an effect of a drug because one has manipulated the cause by design by having one group receive a drug and another a placebo. Here, the users of P-BAT are not manipulating anything by design; instead, users are trying to deduce if someone else—a lab or a distributor—is manipulating something by design. If something is consistently higher or lower than expected, that is evidence that something is amiss—a discrepancy in accounting does not necessarily mean it is fraud, it means regulators should investigate for fraud, and the same is true here.

As seen in Tables 2 and 3, when examining differences by lab, discrepancies reveal themselves (labs X and Y)—this cannot be due to a distributor, as distributor fraud would be consistent across labs (it is not, Table 2 and 3), and not specific to a particular lab (which it is, Tables 2 and 3), thus we have evidence to investigate labs X and Y. Conversely, as seen in Tables 5 and 6 when examining differences by distributor discrepancies reveal themselves (distributor 2)—this cannot be due to a lab, as lab fraud would be consistent across distributors (which it is, seen in Table 6), and not specific to a particular distributor (which it is, seen in Table 5). Thus, we have evidence to investigate distributors. In this way, P-BAT is a type of double ledger leading us to the areas of concern. How a state would proceed with investigation would be particular to and a function of the laws, policies, agencies, and staff of that state and the nature of the discrepancy involved, all of which is outside the scope of the point of this paper, which is to propose a method of detecting a discrepancy in the first place.

The statistics provided here are very basic and are included only to demonstrate how the P-BAT system would work. However, as mentioned, random effects modeling would be needed to analyze these data over time. This paper only provides the design of data collection, P-BAT, not the analysis (except in its most basic form). Aside from statistical practice, how trends are defined and the amount of data that would be needed would be particular to the laws, policies, agencies, and staff of each state. The P-BAT provides a system of data collection so that states will have data with which to make decisions. How lawmakers and regulators make those decisions is outside of the scope of this paper.

Another consideration is the magnitude of fraud. If discrepancies are large, like the examples given here, then very little data over very little time would be needed to have adequate power to detect patterns of concern. However, if discrepancies are very small, then it could take much data over many years to have sufficient power to detect patterns of concern. However, if discrepancies were so small they required a great deal of data, then the question would be how serious the problem is—a question left to regulators to decide and define. Indeed, the hope would be that, over time, after implementing P-BAT, discrepancies would only get smaller and smaller, approaching zero, the goal of quality control.

Although P-BAT uses blind peer-review so that labs effectively police each other and themselves by design, thus making it nearly a self-funded enterprise, given the different state laws, mandates, policies, agencies, and various numbers of staff, stakeholders, and labs that vary state-by-state, the cost of implementing this model will also vary state-by-state based on these factors. However, this cost is then shifted to the labs themselves, unless a state wishes to partially or fully reimburse labs for their tested products, services, and materials.

A final limitation concerns concordant validity in quality control. For example, say a product tested by Lab X passes mold and yeast testing using polymerase chain reaction (PCR). Note, Lab X’s PCR testing passed internal validation testing. However, when the same product is sent to Lab Y, the product fails mold and yeast testing. Lab Y uses plate and count methodology, and Lab Y’s methodology also passed internal validation testing. The fact that using different methods, equipment, and techniques can result in contradictory results concerning safe mold and yeast levels places the point of quality control for cannabis markets into serious question. For decision-making purposes, both cannot be simultaneously “right,” known as concordant validity. That is, if “valid” results can mean both values (i.e., pass and fail) are correct, then why bother to see if they are concordant in the first place (i.e., if pass vs. fail is as valid as pass vs. pass)? This issue concerns the problem of differences in sensitivity and precision between valid testing methods, which should prompt regulators to push toward standardization in the industry for apples-to-apples comparisons.

Therefore, P-BAT is designed to reveal these differences so that states can enact testing standards for apple-to-apple comparisons. However, if states do not require standards for equipment and techniques to be the same, then labs that use equipment and techniques that pass most product will likely be preferred over labs that use equipment and techniques that pass less product; likely, labs using the former will either change their equipment and techniques to pass more product or these labs will go out of business, which may not benefit the end consumers, as indicated by Dr. Land in the WSJ (Armour 2024).

In summary, P-BAT provides a quality assurance framework that disincentivizes “Lab shopping,” “Hero sampling,” poor storage techniques, and product adulteration. It also helps negate the negative impact of the Hawthorne effect on traditional lab testing programs like round robins, PT samples, and spiked samples while incentivizing quality in regards to all the data generated by a particular testing lab as lab directors will be aware of the fact that their whole process is being observed via its outputs. It is transparent, nearly self-funded, and trustless—there is no need to trust the behavior of any one actor or laboratory. Perhaps the main advantage of P-BAT is that if reports from P-BAT were to be made publicly available, then consumers could make informed decisions about their purchases based on the quality data derived from P-BAT. This would further incentivize laboratories to serve and be accountable to the end consumer instead of cultivators and distributors, thus forcing laboratories to continue to compete with each other, but for end consumers, not cultivators and distributors.

#### Notes

*Hero-sampling*. “Hero sampling” refers to practices where a premium sample is selected by either a lab or a cultivators using non-randomized methods that do not adhere to appropriate statistical sampling methods, resulting in the test sample not being adequately representative of the product that will ultimately be sold to the public bearing the inaccurate (and usually inflated) THC concentration levels on its label (Paulson et al. 2022; Schwabe et al. 2023; Smith 2018; Toth 2022). This would become evident if a lab consistently returned a lower THC concentration value during the blinded testing than what was originally reported on the COA.

“*Lab shopping*”. This can be with respect to achieving a higher Total THC number to boost product value or get a product that is contaminated with something that could be detrimental to human health to market. For problems with contaminant analysis, the following should be considered. A lab that consistently returns a pass on a product that other labs are failing would indicate poor methodology that needs to be rectified. Poor methodology or fraudulent activity would be detected if a lab consistently generated a different result for a given sample where they were responsible for the original COA. For THC concentration shopping, if one of the labs consistently generates higher THC concentration numbers across the board, this would indicate potency inflation via an analytical bias or fraudulent means. If a lab is showing a large degree of variance across the board this would help identify poor analytical techniques that need addressing. Regulators in many state markets have begun calling for greater efforts to standardize the analytical practices used by cannabis testing laboratories, and the method proposed here for blinded round robin surveillance may help identify those practices that should be universally adopted across the industry. (Paulson et al. 2022; Schwabe et al. 2023; Smith 2018; Toth 2022).

## Data Availability

NA.

## Abbreviations:

THC: Tetrahydrocannabinol
P-BAT: Peer-review Blinded Assay Test
COA: Certificate Of Analysis
SPC: Statistical Process Control
